# Association between serum uric acid levels and bone mineral density in patients with osteoporosis: a cross-sectional study

**DOI:** 10.1186/s12891-023-06414-w

**Published:** 2023-04-18

**Authors:** Min-zhe Xu, Ke Lu, Xu-feng Yang, Yao-wei Ye, Si-ming Xu, Qin Shi, Ya-qin Gong, Chong Li

**Affiliations:** 1 Department of Orthopedics, Affiliated Kunshan Hospital of Jiangsu University, Suzhou, 215300 Jiangsu China; 2 Department of Orthopedics, Gusu School, Nanjing Medical University, The First People’s Hospital of Kunshan, Suzhou, Jiangsu 215300 China; 3 Department of Orthopedics, the First Affiliated Hospital of Soochow University, Orthopedic Institute of Soochow University, Suzhou, 215031 Jiangsu China; 4 Information Department, Affiliated Kunshan Hospital of Jiangsu University, Suzhou, 215300 Jiangsu China

**Keywords:** Osteoporosis, Uric acid, Bone mineral density, Threshold effect

## Abstract

**Background:**

The results of studies exploring the association between serum uric acid (SUA) and bone mineral density (BMD) have been controversial and inconsistent. We thus sought to explore whether SUA levels were independently associated with BMD in patients with osteoporosis (OP).

**Methods:**

This cross-sectional analysis was conducted using prospectively obtained data from the Affiliated Kunshan Hospital of Jiangsu University database pertaining to 1,249 OP patients that were hospitalized from January 2015 – March 2022. BMD was the outcome variable for this study, while baseline SUA levels were the exposure variable. Analyses were adjusted for a range of covariates including age, gender, body mass index (BMI) and a range of other baseline laboratory and clinical findings.

**Results:**

SUA levels and BMD were independently positively associated with one another in OP patients. Following adjustment for age, gender, BMI, blood urae nitrogen (BUN), and 25(OH)D levels, a 0.0286 g/cm^2^ (β, 0.0286; 95% confidence interval [CI], 0.0193—0.0378, *P* < 0.000001) increase in BMD was observed per 100 μmol/L rise in SUA levels. A non-linear association between SUA and BMD was also observed for patients with a BMI < 24 kg/m^2^, with a SUA level inflection point at 296 μmol/L in the adjusted smoothed curve.

**Conclusions:**

These analyses revealed SUA levels to be independently positively associated with BMD in OP patients, with an additional non-linear relationship between these two variables being evident for individuals of normal or low body weight. This suggests that SUA levels may exert a protective effect on BMD at concentrations below 296 μmol/L in normal- and low-weight OP patients, whereas SUA levels above this concentration were unrelated to BMD.

**Supplementary Information:**

The online version contains supplementary material available at 10.1186/s12891-023-06414-w.

## Background

Osteoporosis (OP) is a form of chronic progressive metabolic disease that results in the aberrant loss of bone mass and concomitant microstructural deterioration within the bone tissue, ultimately increasing the fragility of these bones and increasing the risk of fracture [1]. Low bone mineral density (BMD) was associated with an estimated 438,000 deaths and 16.6 million disability-adjusted life years in 2019, corresponding to respective 111.1% and 93.8% increases relative to 1990. Rising rates of disability and death associated with low BMD have been observed in China, Australia, Canada, and the USA, among other countries [2], and these rising rates of OP have imposed a growing social and economic burden on affected populations [3]. Efforts to define factors that can reduce the risk of OP and improve overall bone health are thus critical to global public health.

Humans exhibit high levels of several different natural antioxidants including albumin, bilirubin, and uric acid. Of these, uric acid is the most abundant, with heterocyclic serum uric acid (SUA) composed of carbon, hydrogen, oxygen, and nitrogen being produced as the final byproduct of human purine metabolism. SUA has been suggested in some reports to have a beneficial impact on BMD [4–8], with its putative benefits being attributed to its robust antioxidant activity in vivo and in vitro [9, 10]. Specifically, the antioxidant activity of SUA enables it to suppress osteoclast-mediated bone resorption and to promote osteoblastogenesis, thus enhancing BMD [11]. Reactive oxygen species (ROS) including hydroxyl radicals and singlet oxygen can contribute to reductions in BMD, and the ability of SUA to mitigate such stress may enable it to play a protective role in OP.

Recent work has sought to explore the association between SUA and BMD, but the results of these studies have been controversial and inconsistent. While some work suggests that high SUA levels can exert a protective impact on BMD [12], other work has revealed no relationship between SUA and BMD in the lumbar spine [13]. Given current uncertainty regarding the relationship between these clinical variables, this study was designed to explore relationships between SUA and BMD in a representative population of hospitalized OP patients.

## Materials and methods

### Study design and subjects

**Fig. 1:**
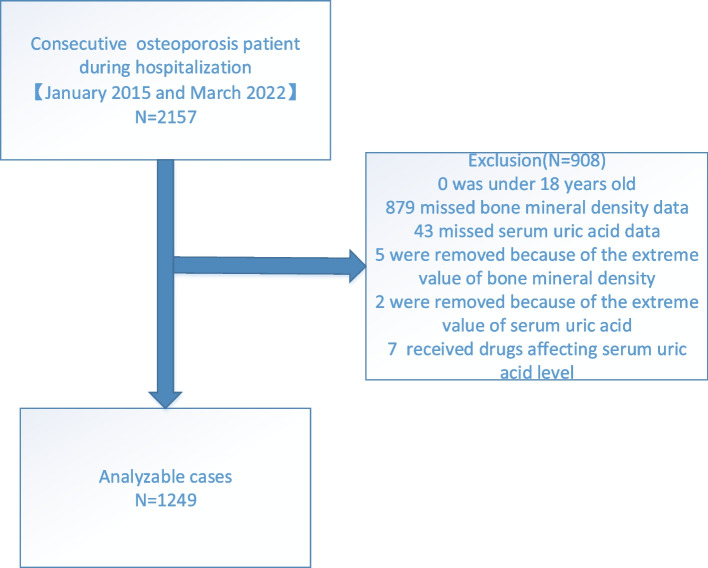
Study flow chart

OP is diagnosed based on the observation of fragility-associated fractures when no other metabolic bone disorders are evident, even when BMD (T-score) is normal, or by a T score ≤ -2.5 for the lumbar spine, femoral neck, total hip, or 1/3 radius (33% radius) even when fractures are not evident. Osteoporotic fractures (OPFs), also referred to as fragility fractures, are caused by low-energy mechanisms, such as falls from standing height or below. The most severe OPFs are hip fractures. Additional low-trauma fractures that are regarded as OPFs include certain distal forearm fractures, proximal humerus fractures, vertebral fractures, and pelvic fractures [14]. OP cases are separated into primary and secondary OP [15]. Postmenopausal, age-related, and idiopathic OP are the three subtypes of primary OP [16]. The term "secondary osteoporosis" refers to bone loss brought on by specific, well-defined clinical diseases [17]. The present study enrolled consecutive hospitalized primary OP patients with definitive diagnoses. In total, 2,157 consecutive hospitalized primary OP patients were evaluated for study inclusion (Fig. 1). Patients were excluded if they were under 18 years old, if their SUA or BMD data were missing, if they exhibited extreme BMD or SUA values, or if they were taking drugs with the potential to impact SUA levels. In total, 1,249 patients were ultimately included in the present analysis based on these criteria. Since Zoledronic acid (ZOL) was being administered to all of these individuals for the first time, the impact of bisphosphonates on BMD may be disregarded. These patients exhibited a mean (± SD) level of 288.61 ± 83.79 μmol/L, and were stratified into four SUA quartiles: Q1, < 231 μmol/L; Q2, 231–278 μmol/L; Q3, 278–341 μmol/L, and Q4, > 341 μmol/L. The Affiliated Kunshan Hospital of Jiangsu University approved this study (approval No. 2020–03-046-K01), which was consistent with the Declaration of Helsinki. Patient data were initially recorded for the improvement of hospital quality, with all analyses being conducted by individuals blinded to patient identity. As this was an observational study and data were gathered anonymously, written informed consent was not required for these analyses. The informed consent was waived by the Ethics Committee of the Affiliated Kunshan Hospital of Jiangsu University.

### Exposure and outcome variables

SUA levels, as measured via an enzymatic colorimetric method before ZOL injection between January 2015 and March 2022, were the exposure variable in this study. Lumbar spine BMD, as measured via dual-energy X-ray absorptiometry (DXA) with a Hologic dual-energy X-ray bone density instrument (Discovery Wi, Hologic Inc, USA), was the outcome variable for this study. The same instrument and the same experienced operator were used to collect all measurements through the use of standardized procedures. Each day prior to participant examination, the machine was subject to standard quality control procedures. Lumbar spine BMD values (g/cm^2^) were based on data from the first, second, third, and fourth lumbar vertebra.

### Covariate analyses

Many different potential covariates were analyzed in the present study, including age, gender, BMI (weight/height^2^; overweight: 24–27.9 kg/m^2^, obese: ≥ 28 kg/m^2^ based on a meta-analysis organized by the Working Group on Obesity in China [18]), Charlson comorbidity index (CCI, [19]) score, primary diagnosis (OP without fractures/OPF), calcitonin use, surgery (yes/no), hemoglobin, monocyte count, lymphocyte count, platelet count, neutrophil count, triglycerides, total cholesterol, albumin, calcium, AST (aspartate aminotransferase), ALT (alanine aminotransferase), creatinine (Cr), blood urea nitrogen (BUN), low-density lipoprotein, high-density lipoprotein, apolipoprotein A, apolipoprotein B, homocysteine, glucose, and 25(OH)D (25-hydroxy vitamin D) levels. All blood samples were collected from fasting patients. Calcitonin use was defined by the intramuscular or subcutaneous administration of 50 IU calcitonin per day. Surgery was defined by hospitalization due to fragility fractures.

### Statistical analyses

Continuous demographic, laboratory, and clinical data are presented as means with standard deviations (SD) or medians (25th, 75th), while categorical data are presented in the form of frequencies (percentages). Pearson’s chi-square tests or Fisher’s exact tests were used for univariate analyses of categorical data, while continuous data were compared using independent sample t-tests and Mann–Whitney U tests when normally and non-normally distributed, respectively. Associations between OP patient characteristics and BMD were also examined through univariate analyses.

Independent relationships between SUA levels and BMD in patients with OP were explored using generalized estimating equations (GEE) with appropriate adjustment for covariates. Developed models included unadjusted and minimally adjusted models (Model 1 and Model 2, respectively) as well as fully-adjusted models (Model 3/4). Initially, variance inflation factor (VIF) analyses were used to detect the collinearity of covariance, after which the decision to adjust for these covariates was made based on the following criteria: (1) a change in the matched odds ratio (OR) of ≥ 10% was observed when the covariate was added to the basic model or removed from the full model; (2) covariates meeting criterion 1 or covariates exhibiting a *P* < 0.1 in univariate models [20]. Model 3 and Model 4 were respectively developed using Criterion 1 and Criterion 2 for covariate adjustment. Finally, four models were established, as follows: Model 1 was unadjusted; Model 2 (minimally adjusted model) was adjusted for age, gender, BMI, and 25(OH)D levels; Model 3 was adjusted for age, gender, BMI, 25(OH)D levels, and BUN; Model 4 was adjusted for age, gender, BMI, 25(OH)D levels, primary diagnosis, BUN, ALT, AST, and Cr.

A generalized additive model (GAM) was used to detect potential non-linear correlative relationships. When such relationships were evident, a two-piecewise linear regression model was used to identify threshold effects for the resultant smoothing curves. A recursive method was used to automatically calculate the inflection point using a maximum likelihood model when these curves exhibited a clear ratio [21]. The robustness of these analyses and their variations among patient subgroups were performed by repeating subgroup analyses when patients were stratified based on particular covariates, with the likelihood ratio test (LRT) being used to analyze subgroup interactions and modifications.

R packages (http://www.R-project.org, The R Foundation) and EmpowerStats (http://www.empowerstats.com, X&Y Solutions, Inc, MA, USA) were used for all analyses, with a two-sided *P* < 0.05 as the significance threshold.

## Results

### Patient characteristics

Baseline characteristics for patients with OP hospitalized from January 2015 – March 2022 (*n* = 1,249) in the established SUA quartiles are summarized in Table 1. These patients (18.65% male, 81.35% female) exhibited an average age of 67.94 ± 9.61 years. The mean BMD for these patients was 0.76 ± 0.14 g/cm^2^, while the mean SUA was 288.61 ± 83.79 μmol/L in the overall patient population. Patients were stratified into SUA quartiles (< 231 μmol/L, 231–278 μmol/L, 278–341 μmol/L, and > 341 μmol/L), and differences in BMD, platelet count, lymphocyte count, albumin, calcium, ALT, AST, Cr, BUN, glucose, triglyceride, high-density lipoprotein, low-density lipoprotein, apolipoprotein A, homocysteine, 25(OH)D levels, gender, BMI, calcitonin use, surgery, and primary diagnosis were evident among these quartiles. Specifically, patients in higher SUA quartiles were more likely to exhibit higher BMD (Q1: 0.71 ± 0.12 g/cm^2^; Q2: 0.75 ± 0.13 g/cm^2^; Q3: 0.78 ± 0.14 g/cm^2^; Q4: 0.80 ± 0.15 g/cm^2^, *P* < 0.001).

**Table 1:** Patient characteristics based on SUA quartiles

Characteristics	Total	Mean** ± **SD / N (%)				*P*-value	*P*-value*
		Q1(< 231 μmol/L)	Q2(231–278 μmol/L)	Q3(278–341 μmol/L)	Q4(> 341 μmol/L)		
N	1249	311	311	313	314		
BMD, g/cm^2^	0.76 ± 0.14	0.71 ± 0.12	0.75 ± 0.13	0.78 ± 0.14	0.80 ± 0.15	< 0.001	< 0.001
Age, y	67.94 ± 9.61	68.28 ± 8.69	66.90 ± 9.61	67.74 ± 9.31	68.83 ± 10.66	0.076	0.053
Platelet count, × 10^9^/L	185.12 ± 61.04	185.08 ± 64.99	191.84 ± 58.52	181.59 ± 53.16	181.98 ± 66.29	0.133	0.045
Neutrophil count, × 10^9^/L	4.47 ± 2.35	4.38 ± 2.12	4.51 ± 2.42	4.35 ± 2.39	4.66 ± 2.44	0.355	0.206
Lymphocyte count, × 10^9^/L	1.41 ± 0.56	1.27 ± 0.50	1.40 ± 0.54	1.48 ± 0.59	1.47 ± 0.58	< 0.001	< 0.001
Monocyte count, × 10^9^/L	0.41 ± 0.19	0.40 ± 0.17	0.41 ± 0.18	0.40 ± 0.20	0.43 ± 0.20	0.168	0.257
Albumin, g/L	40.26 ± 4.75	39.76 ± 4.37	40.61 ± 4.65	40.58 ± 4.69	40.07 ± 5.21	0.070	0.013
Hemoglobin, g/L	124.20 ± 16.72	121.98 ± 18.46	125.07 ± 14.24	125.26 ± 14.39	124.47 ± 19.04	0.055	0.065
Calcium, mmol/L	2.27 ± 0.18	2.24 ± 0.22	2.27 ± 0.15	2.28 ± 0.13	2.28 ± 0.20	0.012	< 0.001
ALT, U/L	21.14 ± 13.59	18.85 ± 10.84	20.56 ± 13.62	21.04 ± 11.96	24.09 ± 16.72	< 0.001	< 0.001
AST, U/L	23.82 ± 10.68	21.99 ± 8.27	23.37 ± 9.82	23.93 ± 9.93	25.96 ± 13.61	< 0.001	0.002
Cr, μmol/L	61.39 ± 24.98	54.87 ± 19.63	55.32 ± 12.87	60.61 ± 18.73	74.66 ± 36.56	< 0.001	< 0.001
CRP, mg/L	1.66 ± 4.60	1.35 ± 3.10	1.75 ± 3.56	1.51 ± 3.49	2.05 ± 7.10	0.760	0.762
BUN, mmol/L	6.24 ± 1.82	5.76 ± 1.45	5.83 ± 1.45	6.33 ± 1.60	7.03 ± 2.33	< 0.001	< 0.001
Glucose, mmol/L	6.06 ± 1.95	6.32 ± 2.14	6.12 ± 2.01	5.88 ± 1.62	5.92 ± 1.96	0.019	0.041
Total cholesterol, mmol/L	4.58 ± 1.01	4.52 ± 1.04	4.71 ± 1.05	4.54 ± 0.95	4.56 ± 0.99	0.166	0.110
Triglyceride, mmol/L	1.38 ± 0.93	1.22 ± 1.12	1.30 ± 0.72	1.37 ± 0.82	1.60 ± 0.98	< 0.001	< 0.001
High-density lipoprotein, mmol/L	1.46 ± 0.33	1.50 ± 0.33	1.52 ± 0.33	1.45 ± 0.35	1.40 ± 0.30	< 0.001	< 0.001
Low-density lipoprotein, mmol/L	2.74 ± 0.81	2.63 ± 0.85	2.80 ± 0.79	2.71 ± 0.78	2.82 ± 0.80	0.056	0.040
Apolipoprotein A, g/L	1.38 ± 0.27	1.38 ± 0.27	1.42 ± 0.29	1.36 ± 0.27	1.35 ± 0.26	0.038	0.030
Apolipoprotein B, g/L	0.90 ± 0.24	0.87 ± 0.25	0.92 ± 0.24	0.89 ± 0.23	0.91 ± 0.23	0.085	0.076
Homocysteine, μmol/L	13.12 ± 6.80	11.34 ± 4.91	12.50 ± 7.03	12.88 ± 5.46	15.44 ± 8.39	< 0.001	< 0.001
25(OH)D levels, ng/mL	20.81 ± 8.19	18.52 ± 7.41	21.25 ± 8.20	20.89 ± 7.81	22.59 ± 8.79	< 0.001	< 0.001
Gender, N (%)						< 0.001	-
Male	233 (18.65%)	25 (8.04%)	46 (14.79%)	64 (20.45%)	98 (31.21%)		
Female	1016 (81.35%)	286 (91.96%)	265 (85.21%)	249 (79.55%)	216 (68.79%)		
BMI categorical, N (%)						< 0.001	-
< 24 kg/m^2^	778 (62.29%)	223 (71.70%)	206 (66.24%)	186 (59.42%)	163 (51.91%)		
24–28 kg/m^2^	358 (28.66%)	75 (24.12%)	85 (27.33%)	100 (31.95%)	98 (31.21%)		
≥ 28 kg/m^2^	113 (9.05%)	13 (4.18%)	20 (6.43%)	27 (8.63%)	53 (16.88%)		
CCI score categorical, N (%)						0.197	-
0	879 (70.38%)	234 (75.24%)	224 (72.03%)	209 (66.77%)	212 (67.52%)		
1–2	194 (15.53%)	47 (15.11%)	39 (12.54%)	53 (16.93%)	55 (17.52%)		
3–4	107 (8.57%)	17 (5.47%)	28 (9.00%)	34 (10.86%)	28 (8.92%)		
≥ 5	69 (5.52%)	13 (4.18%)	20 (6.43%)	17 (5.43%)	19 (6.05%)		
Calcitonin usage, N (%)						0.017	-
No	973 (77.90%)	223 (71.70%)	243 (78.14%)	254 (81.15%)	253 (80.57%)		
Yes	276 (22.10%)	88 (28.30%)	68 (21.86%)	59 (18.85%)	61 (19.43%)		
Surgery, N (%)						< 0.001	-
No	636 (50.92%)	119 (38.26%)	162 (52.09%)	179 (57.19%)	176 (56.05%)		
Yes	613 (49.08%)	192 (61.74%)	149 (47.91%)	134 (42.81%)	138 (43.95%)		
Primary diagnosis, N (%)						< 0.001	-
OP without fractures	771 (61.73%)	151 (48.55%)	193 (62.06%)	213 (68.05%)	214 (68.15%)		
OPF	478 (38.27%)	160 (51.45%)	118 (37.94%)	100 (31.95%)	100 (31.85%)		

*Abbreviations*: *SUA* serum uric acid, *SD* standard deviation, *Q1* first quartile, Q2 second quartile Q3 third quartile, *Q4* fourth quartile, *BMD* bone mineral density, *ALT* alanine aminotransferase, *AST* aspartate aminotransferase, *Cr* creatinine, *CRP* C-reaction protein, *BUN* blood urea nitrogen, *25(OH)D* 25-hydroxy vitamin D, *BMI* body mass index, *CCI* Charlson comorbidity index, *OP* osteoporosis, *OPF* osteoporotic fracture

*P*-value*: Kruskal Wallis Rank Test for continuous variables, Fisher Exact for categorical variables with Expects < 10

### Univariate analyses of factors associated with BMD

In univariate analyses, a clear relationship was observed between BMD and variables including gender, age, BMI, neutrophil count, lymphocyte count, monocyte count, albumin, hemoglobin, ALT, AST, Cr, triglyceride, 25(OH)D levels, calcitonin use, primary diagnosis, and SUA levels (Table 2). No other analyzed variables were related to BMD in these OP patients.

**Table 2:** Univariate analyses of factors associated with BMD

Characteristics	Statistics	β^a^ (95% CI) *P*-value
Gender, N (%)		
Male	233 (18.6549%)	Reference
Female	1016 (81.3451%)	-0.1082 (-0.1274, -0.0891) < 0.000001
Age, y	67.9416 ± 9.6115	-0.0027 (-0.0035, -0.0019) < 0.000001
BMI categorical, N (%)		
< 24 kg/m^2^	778 (62.2898%)	Reference
24–28 kg/m^2^	358 (28.6629%)	0.0495 (0.0322, 0.0669) < 0.000001
≥ 28 kg/m^2^	113 (9.0472%)	0.0650 (0.0376, 0.0923) 0.000004
Platelet count, × 10^9^/L	185.1208 ± 61.0412	-0.0000 (-0.0002, 0.0001) 0.599814
Neutrophil count, × 10^9^/L	4.4744 ± 2.3500	0.0043 (0.0009, 0.0076) 0.012360
Lymphocyte count, × 10^9^/L	1.4060 ± 0.5580	0.0199 (0.0058, 0.0340) 0.005661
Monocyte count, × 10^9^/L	0.4122 ± 0.1880	0.0467 (0.0049, 0.0885) 0.028736
Albumin, g/L	40.2558 ± 4.7470	0.0022 (0.0005, 0.0038) 0.009684
Hemoglobin, g/L	124.2002 ± 16.7161	0.0010 (0.0005, 0.0014) 0.000059
Calcium, mmol/L	2.2704 ± 0.1783	-0.0074 (-0.0514, 0.0365) 0.739645
ALT, U/L	21.1433 ± 13.5909	0.0015 (0.0009, 0.0020) < 0.000001
AST, U/L	23.8199 ± 10.6814	0.0011 (0.0004, 0.0018) 0.003639
Cr, μmol/L	61.3947 ± 24.9772	0.0008 (0.0005, 0.0012) < 0.000001
CRP, mg/L	1.6649 ± 4.6003	0.0029 (-0.0002, 0.0061) 0.068442
BUN, mmol/L	6.2376 ± 1.8168	0.0032 (-0.0011, 0.0075) 0.145362
Glucose, mmol/L	6.0574 ± 1.9495	-0.0004 (-0.0044, 0.0036) 0.854772
Total cholesterol, mmol/L	4.5818 ± 1.0068	0.0020 (-0.0069, 0.0109) 0.660990
Triglyceride, mmol/L	1.3796 ± 0.9285	0.0099 (0.0002, 0.0196) 0.044713
High-density lipoprotein, mmol/L	1.4647 ± 0.3296	-0.0236 (-0.0508, 0.0037) 0.090335
Low-density lipoprotein, mmol/L	2.7438 ± 0.8069	0.0089 (-0.0023, 0.0200) 0.118669
Apolipoprotein A, g/L	1.3769 ± 0.2728	-0.0105 (-0.0435, 0.0224) 0.530965
Apolipoprotein B, g/L	0.8986 ± 0.2364	0.0122 (-0.0258, 0.0502) 0.529786
Homocysteine, μmol/L	13.1186 ± 6.8044	0.0010 (-0.0003, 0.0023) 0.126096
25(OH)D levels, ng/mL	20.8138 ± 8.1942	0.0026 (0.0017, 0.0036) < 0.000001
CCI score categorical, N (%)		
0	879 (70.3763%)	Reference
1–2	194 (15.5324%)	-0.0172 (-0.0391, 0.0047) 0.124098
3–4	107 (8.5669%)	-0.0048 (-0.0331, 0.0235) 0.740577
≥ 5	69 (5.5244%)	-0.0124 (-0.0469, 0.0221) 0.481759
Calcitonin usage, N (%)		
No	973 (77.9023%)	Reference
Yes	276 (22.0977%)	-0.0259 (-0.0447, -0.0071) 0.007011
Surgery, N (%)		
No	636 (50.9207%)	Reference
Yes	613 (49.0793%)	0.0008 (-0.0149, 0.0164) 0.921790
Primary diagnosis, N (%)		
OP without fractures	771 (61.7294%)	Reference
OPF	478 (38.2706%)	-0.0334 (-0.0494, -0.0175) 0.000044
Serum uric acid, per 100 μmol/L increase	2.8861 ± 0.8379	0.0440 (0.0350, 0.0530) < 0.000001
Serum uric acid quartile, N (%)		
Q1(< 232 μmol/L)	311 (24.8999%)	Reference
Q2(232–280 μmol/L)	311 (24.8999%)	0.0327 (0.0113, 0.0542) 0.002863
Q3(280–336 μmol/L)	313 (25.0600%)	0.0677 (0.0462, 0.0891) < 0.000001
Q4(> 336 μmol/L)	314 (25.1401%)	0.0924 (0.0710, 0.1139) < 0.000001

*Abbreviations*: *BMD* bone mineral density, *CI* confidence interval, *BMI* body mass index, *ALT* alanine aminotransferase, *AST* aspartate aminotransferase, *Cr* creatinine, *CRP* C-reaction protein, *BUN* blood urea nitrogen, *CCI* Charlson comorbidity index, *25(OH)D* 25-hydroxy vitamin D, *OP* osteoporosis, *OPF* osteoporotic fracture

^a^Dependent variable BMD, as a result of univariate analyses for BMD

### Exploration of the association between SUA levels and BMD

Four models were next used to examine the relationship between SUA and BMD in OP patients (Table 3). A clear relationship between these variables was evident in the unadjusted Model 1 (β = 0.0440, 95% CI: 0.0350 to 0.0530, *P* < 0.000001). Model 2, which was adjusted for age, gender, BMI, and 25(OH)D levels, exhibited a similar association (β = 0.0288, 95% CI: 0.0199 to 0.0377, *P* < 0.000001). A positive relationship was also evident for Model 3 (β = 0.0286, 95% CI: 0.0193 to 0.0378, *P* < 0.000001) following adjustment for age, gender, BMI, 25(OH)D levels, and BUN. Model 4, which was adjusted for age, gender, BMI, 25(OH)D levels, primary diagnosis, BUN, ALT, AST, and Cr, also yielded a similar relationship between these variables (β = 0.0235, 95% CI: 0.0139 to 0.0330, *P* = 0.000002).

**Table 3:** Association between SUA levels and BMD in different models

	Model 1^a^ *N* = 1249 β (95%CI) *P-*value	Model 2^b^ *N* = 1249 β (95%CI) *P-*value	Model 3^c^ *N* = 1249 β (95%CI) *P-*value	Model 4^d^ *N* = 1249 β (95%CI) *P-*value
Serum uric acid per 100 μmol/L increase	0.0440 (0.0350, 0.0530) < 0.000001	0.0288 (0.0199, 0.0377) < 0.000001	0.0286 (0.0193, 0.0378) < 0.000001	0.0235(0.0139, 0.0330) 0.000002
Serum uric acid quartile				
Q1(< 231 μmol/L)	Reference	Reference	Reference	Reference
Q2(231–278 μmol/L)	0.0327 (0.0113, 0.0542) 0.002863	0.0163 (-0.0038, 0.0365) 0.112425	0.0163 (-0.0039, 0.0364) 0.114381	0.0135 (-0.0066, 0.0337) 0.187813
Q3(278-341 μmol/L)	0.0677 (0.0462, 0.0891) < 0.000001	0.0451 (0.0248, 0.0653) 0.000014	0.0445 (0.0241, 0.0649) 0.000020	0.0391 (0.0185, 0.0596) 0.000199
Q4(> 341 μmol/L)	0.0924 (0.0710, 0.1139) < 0.000001	0.0558 (0.0348, 0.0769) < 0.000001	0.0547 (0.0331, 0.0763) < 0.000001	0.0428 (0.0207, 0.0650) 0.000153
*P*-value for trend	< 0.000001	< 0.000001	< 0.000001	0.000034

*Abbreviations*: *SUA* serum uric acid, *BMD* bone mineral density, *CI* confidence interval, *Q1* first quartile, *Q2* second quartile, *Q3* third quartile, *Q4* fourth quartile, *BMI* body mass index, *25(OH)D* 25-hydroxy vitamin D, *BUN* blood urea nitrogen, *ALT* alanine aminotransferase, *AST* aspartate aminotransferase, *Cr* creatinine

^a^No adjustment

^b^Adjusted for age, gender, BMI, 25(OH)D levels

^c^Adjusted for age, gender, BMI, 25(OH)D levels, BUN

^d^ Adjusted for age, gender, BMI, 25(OH)D levels, primary diagnosis, BUN, ALT, AST, Cr

SUA levels were used to group patients into quartiles, revealing average BMD values that were 0.0163 g/cm^2^, 0.0445 g/cm^2^, and 0.0547 g/cm^2^ units higher in Q2, Q3, and Q4 relative to Q1 in Model 3. Marked increases in BMD were evident for OP patients in SUA quartiles 3 and 4 under all four models, and in Model 1, BMD levels were higher in Q2 relative to Q1.

To confirm the robustness of Model 3, subgroup analyses were further conducted in which OP patients were stratified according to age, gender, BMI, 25(OH)D levels, and BUN, with analyses being adjusted for the remaining covariates not used for stratification. A highly consistent pattern was observed across these results without any apparent stratification-related interactions (all *P* > 0.05, Table S1). As shown in Fig. S1, relationships between SUA and BMD were consistent in both males and females when adjusting for age, BMI, 25(OH)D levels, and BUN.

### Spline smoothing plot and threshold analyses

**Fig. 2:**
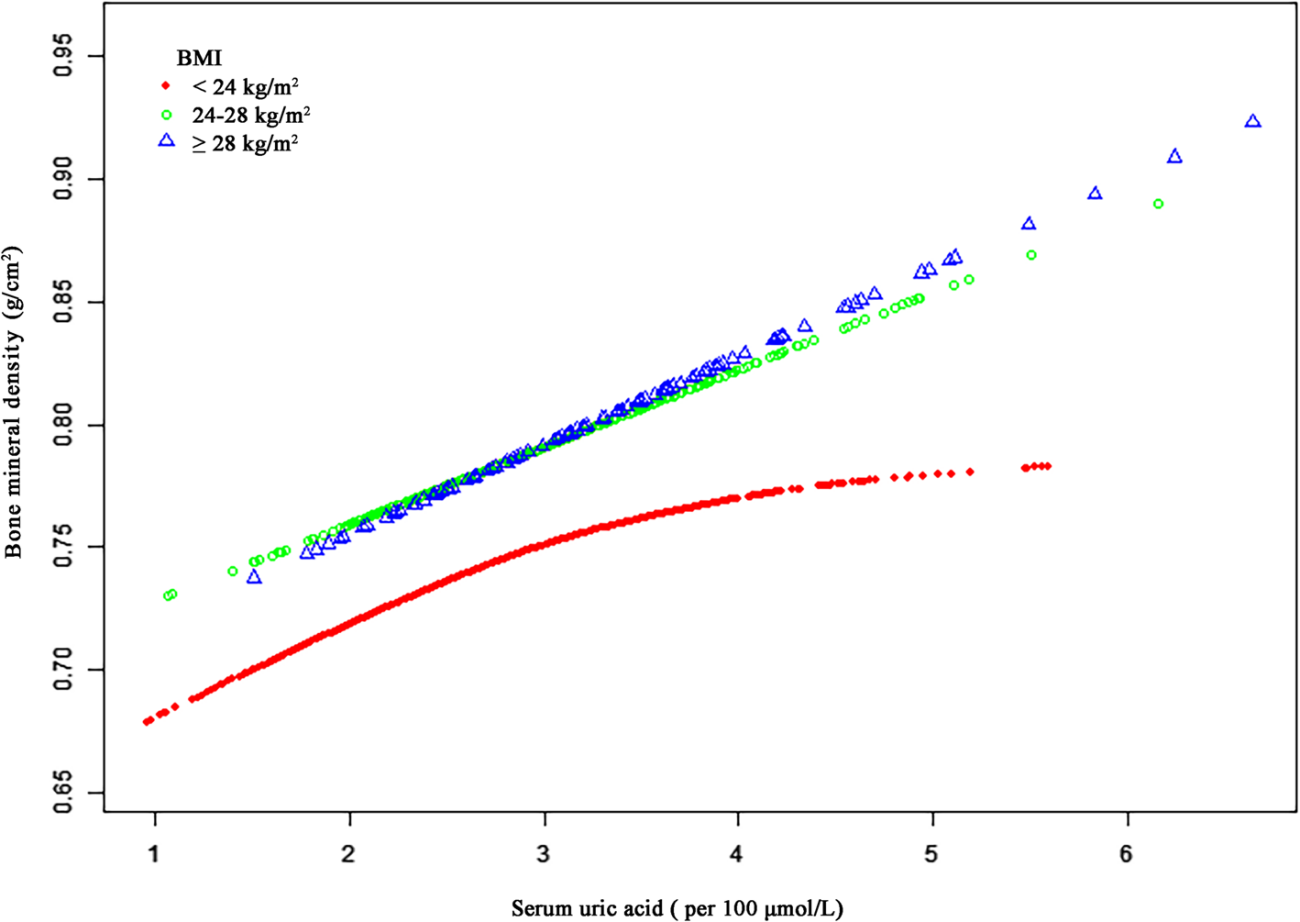
The relationship between SUA and BMD. Adjusted smoothed curves corresponding to the relationship between SUA levels and BMD. A generalized additive model revealed a thresholded non-linear relationship between SUA and BMD in OP patients with a BMI < 24 kg/m2. Red, green, and blue curves respectively correspond to estimated values for OP patients with a BMI < 24 kg/m2, 24–28 kg/m2, and > 28 kg/m2. Models were adjusted for age, gender, 25(OH)D levels, and BUN. The red curve in Model 3 exhibited an inflection point (K) at 2.96 per 100 μmol/L. SUA, serum uric acid; BMD, bone mineral density; BMI, body mass index; 25(OH)D, 25-hydroxy vitamin D; BUN, blood urea nitrogen

A graphical approach was next used to represent estimated exposure–response plots for OP patients stratified according to BMI status in an effort to gauge whether the relationship between SUA levels and BMD was linear or non-linear (Fig. 2). GAM estimation suggested the existence of a non-linear relationship between SUA and BMD for patients with a BMI < 24 kg/m^2^ (*P*-value for LRT = 0.034) following adjustment for age, gender, 25(OH)D levels, and BUN (Table 4). In these normal- and low-weight patients with OP, a threshold non-linear association was detected between SUA levels and BMD, with an inflection point (K = 2.96) being established with a piecewise linear regression model. To the left of this inflection point, the respective effect size, 95% CI, and *P-*values were 0.0438, 0.0217—0.0658, and 0.0001. To the right of this inflection point, SUA levels were unrelated to BMD (β = 0.0029, 95%CI: -0.0200 to 0.0259, *P* = 0.8024). A positive linear relationship was nonetheless observed between SUA and BMD for patients with a BMI of 24–28 kg/m^2^ or ≥ 28 kg/m^2^ (*P*-value for LRT = 0.590,* P*-value for LRT = 0.498).

**Table 4:** Threshold analyses examining the relationship between SUA levels and BMD

	Model 3^a^				
BMI	< 24 kg/m^2^ β (95% CI) *P*-value	24–28 kg/m^2^ β (95% CI) *P*-value	≥ 28 kg/m^2^ β (95% CI) *P*-value	Total	
Model A^b^					P-interaction: 0.673
One line effect	0.0239 (0.0118, 0.0361) 0.0001	0.0291 (0.0116, 0.0466) 0.0012	0.0500 (0.0251, 0.0749) 0.0001		0.0286 (0.0193, 0.0378) < 0.0001
Model B^c^					P-interaction: 0.499
Serum uric acid turning point (K), per 100 μmol/L	2.96	1.91	4.98		2.94
< K	0.0438 (0.0217, 0.0658) 0.0001	-0.0129 (-0.1679, 0.1422) 0.8709	0.0437 (0.0124, 0.0750) 0.0073		0.0413 (0.0229, 0.0598) < 0.0001
> K	0.0029 (-0.0200, 0.0259) 0.8024	0.0309 (0.0122, 0.0495) 0.0013	0.0827 (-0.0180, 0.1834) 0.1106		0.0187 (0.0032, 0.0342) 0.0184
Slope 2 – Slope 1	-0.0408 (-0.0788, -0.0029) 0.0352	0.0437 (-0.1167, 0.2041) 0.5936	0.0390 (-0.0775, 0.1555) 0.5130		-0.0226 (-0.0511, 0.0058) 0.1186
BMD value at K, g/cm^2^	0.7621 (0.7453, 0.7788)	0.7491 (0.7241, 0.7741)	0.8663 (0.8097, 0.9229)		0.7748 (0.7621, 0.7875)
LRT test^d^	**0.034**	0.590	0.498		0.117

*Abbreviations*: *SUA* serum uric acid, *BMD* bone mineral density, *CI* confidence interval, *BMI* body mass index, *25(OH)D* 25-hydroxy vitamin D, *BUN* blood urea nitrogen, *LRT* logarithmic likelihood ratio test

^a^Adjusted for age, gender, 25(OH)D levels and BUN

^b^Linear analysis, *P*-value < 0.05 indicates a linear relationship

^c^Non-linear analysis

^d^*P*-value < 0.05 means Model B is significantly different from Model A, which indicates a non-linear relationship

## Discussion

Here, a cross-sectional analysis of 1,249 hospitalized OP patients revealed a significant positive correlation between SUA levels and BMD. Moreover, adjusted models revealed a non-linear association between these levels in individuals with a BMI < 24 kg/m^2^, exhibiting an inflection point at 296 μmol/L of SUA among BMI. As such SUA levels may have a protective influence on BMD in certain settings, with BMI having the potential to influence the relationship between these two variables. While elevated SUA levels may offer some benefit to the BMD of OP patients, in patients with a BMI of less than 24 kg/m^2^, SUA levels above 296 μmol/L may be unrelated to BMD.

Several epidemiological studies have explored the relationship between SUA and BMD, but the results have been inconsistent. Some reports suggest that SUA is significantly related to BMD and may have a protective impact on bone metabolism when assessing healthy adults [22], type 2 diabetes patients [23, 24], postmenopausal women [8, 25, 26], and elderly individuals [27]. However, some studies have demonstrated that there is no association between SUA and BMD in adult males in the USA [13], postmenopausal women [28], a rodent model of chronic mild hyperuricemia [29], or postmenopausal patients with type 2 diabetes [30]. These inconsistencies may be due to differences in the study populations. Our study found that SUA levels and BMD are independently positively associated, and we specifically focused on patients diagnosed with OP.

The mechanistic basis that links SUA to bone metabolism remains poorly defined, with some research attributing the protective benefits of SUA to its antioxidant activity. Indeed, SUA is an endogenous antioxidant, particularly under conditions of oxidative stress [9], and can readily scavenge free radicals in the plasma. In their review, Lin et al. determined that normal or elevated SUA concentrations were significantly related to reductions in BMD and exhibited protective effects against bone fracture [31]. ROS can readily suppress the differentiation of osteoblasts while enhancing osteoclastic differentiation and activation, ultimately leading to osteopenia [32]. SUA is associated with dose-dependent reductions in osteoclastogenesis and can suppress ROS production in osteoclast precursors [6]. UA also reportedly promotes human bone mesenchymal stem cell proliferation and osteoblastic differentiation while inhibiting the adipogenic differentiation of these cells [33]. In mice treated with oxonic acid, SUA exerts protective efficacy given that these animals exhibit reduced synovial infiltration by inflammatory cells, with corresponding decreases in synovial hyperplasia, bone erosion, and cartilage damage as compared to control animals [34]. Other factors may also explain the observed relationship between SUA and BMD, with muscle mass as one potential mediator of this relationship through processes related to muscle-derived cytokine production and mechanical loading [35]. There is thus a clear need for further studies exploring the underlying mechanisms governing this relationship to firmly establish the value of SUA as a diagnostic biomarker associated with OP and other forms of disease. SUA exhibits paradoxical concentration-dependent effects, exhibiting beneficial antioxidant activity at normal concentrations but serving as a metabolic syndrome risk factor in the context of hyperuricemia [36]. A randomized controlled trial of postmenopausal women found that while supplemental inosine intake resulted in sustained serum urate concentrations over 6 months, it had no impact on bone turnover-related markers, in contrast with the concept that urate directly influences this bone turnover process [37].

In this study, a threshold effect was observed for the relationship between SUA levels and BMD in patients with a BMI < 24 kg/m^2^. This finding is distinct from that of prior reports and suggests that in normal and lower-weight individuals, it may be important to maintain SUA levels within a desirable range (threshold: 296 μmol/L). Explaining the mechanistic basis for this phenomenon is difficult. We sought to explore differences in the association between SUA and BMD in different patient BMI subgroups. A positive association between BMI and BMD has been reported previously. BMI or weight can affect BMD as a result of the load factor [38]. According to Dalbeth et al., individuals with a high BMI exhibit reduced renal clearance after consuming dietary purines and have a larger renal capacity for UA reabsorption when fasting, with a higher BMI thus being associated with hyperuricemia [39]. It is thus possible that the positive relationship between UA and BMD is a consequence of obesity or higher BMI. However, one study found that ~ 25% of the effect of UA on BMD may be explained by BMI. While this BMI-mediated effect is statistically significant, a large portion of the role of UA in this context is thus not BMI-dependent [23]. In those patients with normal or lower weight, BMI-mediated effects may be less pronounced than in overweight or obese individuals. This may explain the reason why a threshold effect was only observed in patients with a BMI < 24 kg/m^2^.

A recent review indicated that SUA levels can contribute to a higher risk of fracture in patients suffering from hyperuricemia or gouty arthritis, as the combination of oxidative stress and inflammatory cytokine production in response to SUA degradation simultaneously enhanced bone resorption while suppressing bone formation [31]. Antioxidative compounds can thus potentially undergo conversion into deleterious pro-oxidative compounds in certain contexts. Other work has also suggested that pro-oxidative SUA can cause damage in other disease-related settings [40]. Given this apparent paradoxical conflict between the harmful and beneficial effects of SUA, caution is necessary when translating these results to the clinic. While the correction of hyperuricemia is important to lower the risk of other types of disease, it is important to guard against overtreatment which has the potential to mitigate the beneficial impact of SUA as a suppressor of oxidative stress. An individualized determination of optimal SUA levels should instead be made following the assessment of individual patient risk of OP and other diseases. The present results suggest that the BMI of patients should similarly be taken into consideration in this setting. Marin-Mio et al. reported a lower risk of OP in individuals that maintain a healthy muscle mass [41]. In the present analysis, SUA levels > 296 μmol/L were found to have little impact on BMD in patients with a BMI < 24 kg/m^2^, potentially explaining why some prior analyses have failed to detect any relationship between these two clinical variables.

The results of this analysis may have important clinical implications. For one, the observed positive relationship between SUA levels and BMD suggests that higher levels are not necessarily beneficial, particularly among normal- and low-weight patients with OP beyond the identified threshold. This threshold may thus be of value in guiding SUA-focused clinical interventions and can aid in the formulation of appropriate treatment guidelines and clinical procedures for particular subsets of OP patients. Secondly, these results suggest that baseline SUA levels offer predictive value when assessing BMD in OP patients such that these levels have the potential to be incorporated into panels of fracture risk predictors in the context of patient clinical evaluation.

This study exhibits several important strengths. For one, the study population was rigorously screened. Additionally, the relationship between SUA levels and BMD was rigorously examined using four different models that were adjusted for a range of different potential confounding variables including age, gender, BMI, 25(OH)D levels BUN, ALT, AST, Cr, and primary diagnosis. Moreover, differences in the association between SUA and BMD were observed in patients with a BMI < 24 kg/m^2^, potentially explaining prior controversy with respect to the relationship between these two variables in other studies.

There are some limitations to this analysis. For one, while SUA levels were found to be related to BMD in these patients, this does not offer any evidence of causality pertaining to this relationship. Moreover, other potentially relevant biochemical indicators including parathyroid hormone (PTH) and plasma phosphate levels were not analyzed in these patients and have the potential to impact bone metabolism. Markers of bone turnover such as procollagen type I N-terminal propeptide (P1NP) and cross linked C-telopeptide of type I collagen (CTX1) were similarly not analyzed. Third, this was a single-center study of a relatively small patient population such that these findings may not be generalizable to individuals of other ethnicities. In light of these limitations, further large-scale follow-up studies incorporating additional biochemical markers, multi-ethnic populations, a multi-center randomized design will be critical to ensure that these results are robust and replicable. Future studies can additionally focus on the role of BMI in the relationship between SUA and BMD and the mechanisms underlying this relationship.

## Conclusions

In summary, the results of this analysis indicate that SUA levels and BMD are independently positively associated with one another in patients with OP. A non-linear relationship between SUA levels and BMD was also observed in patients with both normal and low body weight, suggesting that these SUA levels may offer protective value for BMD in both normal- and low-weight individuals with OP. Specifically, while SUA levels below 296 μmol/L were predicted to be protective, values above these levels were not associated with BMD. However, additional follow-up research with a larger number of patients will be critical to validate these findings.

## Supplementary Information

**Additional file 1.**

**Additional file 2.**

## Data Availability

The data that support the findings of this study are available from the corresponding author upon reasonable request.

## Abbreviations

SUA: Serum uric acid
BMD: Bone mineral density
BMI: Body mass index
CI: Confidence interval
OP: Osteoporosis
ROS: Reactive oxygen species
ZOL: Zoledronic acid
DXA: Dual-energy X-ray absorptiometry
CCI: Charlson comorbidity index
AST: Aspartate aminotransferase
ALT: Alanine aminotransferase
Cr: Creatinine
BUN: Blood urea nitrogen
25(OH)D: 25-Hydroxy vitamin D
SD: Standard deviations
GEE: Generalized estimating equations
VIF: Variance inflation factor
OR: Odds ratio
GAM: Generalized additive model
LRT: Likelihood ratio test
