# Supplementary material for: Association between serum uric acid levels and bone mineral density in patients with osteoporosis: a cross-sectional study
Source: BMC Musculoskelet Disord. 2023 Apr 18;24:306. doi: 10.1186/s12891-023-06414-w (PMC10111842; doi:10.1186/s12891-023-06414-w)
Supplement: Supplementary file 1 — Additional file 1. [file 12891_2023_6414_MOESM1_ESM.pdf]

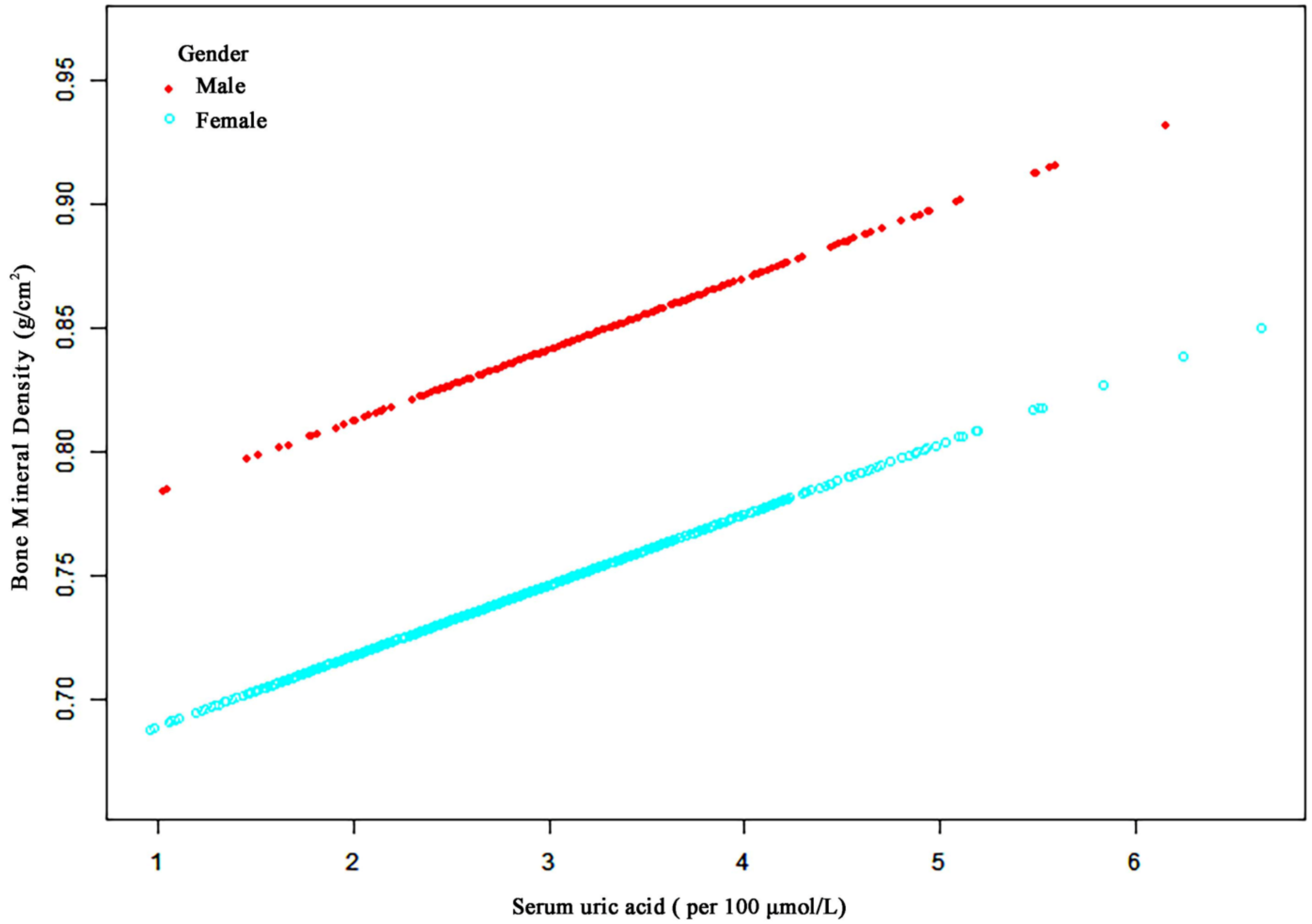

Fig. S1 The relationship between serum uric acid and bone mineral density stratified by gender. Adjusted smoothed curves corresponding to the relationship between SUA levels and BMD. Linear relationships between SUA and BMD were observed for different genders when using a generalized additive model. Red and blue curves respectively correspond to male and female OP patients. Models were adjusted for age, BMI, 25(OH)D levels, and BUN. BMD, bone mineral density; BMI, body mass index; 25(OH)D, 25-hydroxy vitamin D; BUN, blood urea nitrogen.
