# Supplementary material for: Association between serum uric acid levels and bone mineral density in patients with osteoporosis: a cross-sectional study
Source: BMC Musculoskelet Disord. 2023 Apr 18;24:306. doi: 10.1186/s12891-023-06414-w (PMC10111842; doi:10.1186/s12891-023-06414-w)
Supplement: Supplementary file 2 — Additional file 2. [file 12891_2023_6414_MOESM2_ESM.docx]

Table S1. Subgroup analyses exploring the association between serum uric acid levels and BMD

| Subgroup | N | β (95%CI) *P-*value | *P-*value for interaction |
| --- | --- | --- | --- |
| Gender |  |  | 0.9790 |
| Male | 233 | 0.0373 (0.0150, 0.0597) 0.0012 |  |
| Female | 1016 | 0.0275 (0.0176, 0.0374) <0.0001 |  |
| BMI |  |  | 0.6730 |
| < 24 kg/m^2^ | 778 | 0.0239 (0.0118, 0.0361) 0.0001 |  |
| 24-28 kg/m^2^ | 358 | 0.0291 (0.0116, 0.0466) 0.0012 |  |
| ≥ 28 kg/m^2^ | 113 | 0.0500 (0.0251, 0.0749) 0.0001 |  |
| 25(OH)D levels |  |  | 0.6539 |
| Deficiency (<12 ng/mL) | 141 | 0.0414 (0.0204, 0.0624) 0.0002 |  |
| Inadequacy (12-20 ng/mL) | 474 | 0.0272 (0.0125, 0.0419) 0.0003 |  |
| Adequacy (≥20 ng/mL) | 634 | 0.0270 (0.0130, 0.0410) 0.0002 |  |
| Age tertile |  |  | 0.2268 |
| Low | 361 | 0.0257 (0.0071, 0.0442) 0.0070 |  |
| Middle | 457 | 0.0288 (0.0142, 0.0434) 0.0001 |  |
| High | 431 | 0.0313 (0.0159, 0.0467) <0.0001 |  |
| BUN tertile |  |  | 0.9781 |
| Low | 416 | 0.0336 (0.0159, 0.0514) 0.0002 |  |
| Middle | 410 | 0.0213 (0.0048, 0.0378) 0.0118 |  |
| High- | 423 | 0.0289 (0.0151, 0.0426) <0.0001 |  |

Adjusted for age, gender, BMI, 25(OH)D levels and BUN except the subgroup variable.

Abbreviations: BMD, bone mineral density; BMI, body mass index; 25(OH)D, 25-hydroxy vitamin D; BUN, blood urea nitrogen
